# The Effects of Tai Chi Practice on Intermuscular Beta Coherence and the Rubber Hand Illusion

**DOI:** 10.3389/fnhum.2016.00037

**Published:** 2016-02-16

**Authors:** Catherine E. Kerr, Uday Agrawal, Sandeep Nayak

**Affiliations:** ^1^Alpert Medical School, Brown University, ProvidenceRI, USA; ^2^Division of Biology and Medicine, Brown University, ProvidenceRI, USA

**Keywords:** Tai Chi, beta rhythm, intermuscular coherence, EMG, body awareness, rubber hand illusion, embodiment

## Abstract

Tai Chi (TC) is a slow-motion contemplative exercise that is associated with improvements in sensorimotor measures, including decreased force variability, enhanced tactile acuity, and improved proprioception, especially in elderly populations. Here, we carried out two studies evaluating the effect of TC practice on measures associated with sensorimotor processing. In study 1, we evaluated TC’s effects on an oscillatory parameter associated with motor function, beta rhythm (15–30 Hz) coherence, focusing specifically on beta rhythm intermuscular coherence (IMC), which is tightly coupled to beta corticomuscular coherence (CMC). We utilized electromyography (EMG) to compare beta IMC in older TC practitioners with age-matched controls, as well as novices with advanced TC practitioners. Given previous findings of elevated, maladaptive beta coherence in older subjects, we hypothesized that increased TC practice would be associated with a monotonic decrease in beta IMC, but rather discovered that novice practitioners manifested higher beta IMC than both controls and advanced practitioners, forming an inverted U-shaped practice curve. This finding suggests that TC practice elicits complex changes in sensory and motor processes over the developmental lifespan of TC training. In study 2, we focused on somatosensory (e.g., tactile and proprioceptive) responses to the rubber hand illusion (RHI) in a middle-aged TC group, assessing whether responses to the illusion became dampened with greater cumulative practice. As hypothesized, TC practice was associated with decreased likelihood to misattribute tactile stimulation during the RHI to the rubber hand, although there was no effect of TC practice on measures of proprioception or on subjective reports of ownership. These studies provide preliminary evidence that TC practice both modulates beta network coherence in a non-linear fashion, perhaps as a result of the focus on not only efferent motor but also afferent sensory activity, and alters tactile sensations during the RHI. This work is the first to show the effects of TC on low level sensorimotor processing and integrated body awareness, and this multi-scale finding may help to provide a mechanistic explanation for the widespread sensorimotor benefits observed with TC practice in symptoms associated with aging and difficult illnesses such as Parkinson’s disease.

## Introduction

A growing number of studies have reported that mindfulness meditation elicits health benefits, including enhanced attentional processing (Jha et al., 2007; MacLean et al., 2010), cognition (Jha et al., 2010; Mrazek et al., 2013), and sensory processing (Mirams et al., 2013). An important feature of mindfulness is the cultivation of somatically directed attention, as individuals are instructed to attend to their sensory experience, such as the sensations of the flow of the breath (Kerr et al., 2013). In tandem with these behavioral findings, previous work from our lab and others has demonstrated that training in mindfulness leads to enhanced attentional control over excitability in the primary somatosensory cortex map of the body, as indexed by dynamic control over alpha (7–14 Hz) rhythms in this region (Kerr et al., 2011).

Tai Chi (TC) is a less well studied contemplative exercise in which practitioners cultivate attention to body sensations during slow movement and static warm-up postures (Wayne and Fuerst, 2013). Derived from a longstanding Chinese martial arts practice, TC is associated with enhancements in both motor and sensory domains, especially in elderly populations. In addition to improved balance and decreased fall risk in the elderly (Wolf et al., 1997; Schleicher et al., 2012), reduced force variability during complex motor tasks (Christou et al., 2003), and efficacy in reducing symptoms related to Parkinson’s disease (Li et al., 2012), TC also trains somatosensory perceptual capacities such as proprioception (Li et al., 2008) and tactile acuity in the fingertip (Kerr et al., 2008) and the foot (Richerson and Rosendale, 2007).

Given TC’s emphasis on somatic attention during movement, the observed benefits associated with the practice, and considering our previous finding that mindfulness modulates somatosensory alpha oscillations, here we sought to investigate the effects of TC practice on a sensorimotor parameter, beta (15–30 Hz) oscillatory rhythm coherence, that is related to the sensorimotor alpha rhythm recorded over sensorimotor cortex (Jones et al., 2009).

The beta band signal descending from motor cortex to the muscles is thought to play a causal role in synchronizing neural activity both across cortex and the muscles (Engel and Fries, 2010). This signal is referred to as beta corticomuscular coherence (CMC). The signal can also be captured intermuscularly, in recordings of opponent muscles involved in a static, isometric task (i.e., beta intermuscular coherence, IMC; Kilner et al., 1999; Baker, 2007). Beta CMC/IMC coherence during an isometric task is thought to temporally align the muscles and regulate force variability (Witte et al., 2007). In fact, beta CMC has been found to be inversely correlated with force variability (Johnson and Shinohara, 2012) in normal-aged subjects. However, in aging populations, beta CMC becomes over-expressed and loses its functional relationship to force variability (Johnson and Shinohara, 2012). In other words, although the descending beta CMC signal seems to serve a motor function of reducing force variability in younger populations, this functionality is absent in elderly populations as a consequence of becoming chronically elevated. A further complexity arises from the fact that in a small number of studies beta CMC has been associated with the afferent flow of proprioceptive information from the muscles to the cortex (Baker et al., 2006), although the functional significance of this finding remains unclear.

Given that beta IMC is tightly coupled with beta CMC (van Ede and Maris, 2013), here we evaluated the effects of TC practice on beta IMC. We hypothesized that TC practice, which has demonstrated sensory and motor benefits in the elderly, would reduce maladaptive age-related increases in muscle network coherence in a population of elderly TC practitioners. Furthermore, we hypothesized that this reduction in beta IMC in a sample of elderly TC practitioners would restore the inverse relationship between force variability and beta coherence found in younger populations. To test these hypotheses, we recorded beta IMC during a low force isometric task in a group of elderly TC practitioners and age-matched elderly controls.

As an exploratory extension of the primary study, we also investigated the effect of TC practice on the rubber hand illusion (RHI), a paradigm designed to test multi-sensory integration via experimental manipulation of visual, tactile, and proprioceptive streams of information (Botvinick and Cohen, 1998). In brief, the RHI involves an experimenter synchronously or asynchronously stroking both the real hand of the subject which is hidden from view, as well as a fake rubber hand placed in front of the subject in clear view. Often, subjects report experiencing altered tactile sensations, as if the sensations were originating from the strokes seen on the rubber hand, and numerous studies report that during the RHI, somatosensory processing is altered (Moseley et al., 2008; Zopf et al., 2011; Zeller et al., 2015). In addition, behavioral measures such as proprioceptive drift, which measures the distance from the perceived location of one’s hand to its actual location, have indicated that the illusion induces a perceptual bias toward the concealed, rubber hand [although this phenomena is not always reproducible (Rohde et al., 2011; Abdulkarim and Ehrsson, 2015)].

Beyond tactile and proprioceptive components of the illusion, the RHI is widely used to study body-ownership and multi sensory processing underlying perception of one’s own-body in healthy [see review (Kilteni et al., 2015)], as well as clinical populations [e.g., eating disorders (Eshkevari et al., 2012), schizophrenia (Thakkar et al., 2011), and chronic pain (Moseley et al., 2012)]. Given the phenomenological reports in the TC literature of changes in practitioners in the experience of embodiment (Wayne, 2013), we specifically utilized the RHI to investigate long term effects of TC practice on tactile and proprioceptive changes associated with the practice as well as embodiment as in (Longo et al., 2008b). We hypothesized that since TC practice enhances tactile acuity (Kerr et al., 2008) and improved tactile acuity is associated with decreased misattribution of tactile sensations to the fake rubber hand (Longo et al., 2008a), the TC practitioners in our sample will more accurately be able to attribute tactile sensations to the real arm. Furthermore, because TC enhances proprioception (Li et al., 2008; Longo et al., 2008a), we expected the TC practitioners in our sample to more accurately identify their hand position and thus show decreased proprioceptive drift. In addition, we hypothesized that this decreased perceptual error in tactile acuity and proprioception would result in a reduction of perceived illusory ownership of the rubber hand, as this aspect of the illusion arises from a multisensory integration of vision, proprioception, and touch. Note: the sample of TC practitioners in study 2, recruited from the same TC studio as study 1, was composed of a significantly younger (*p* < 0.002) cohort of practitioners (64.3 ± 4.7 in study 1; 51.5 ± 14.1 years in study 2).

**FIGURE 1:**
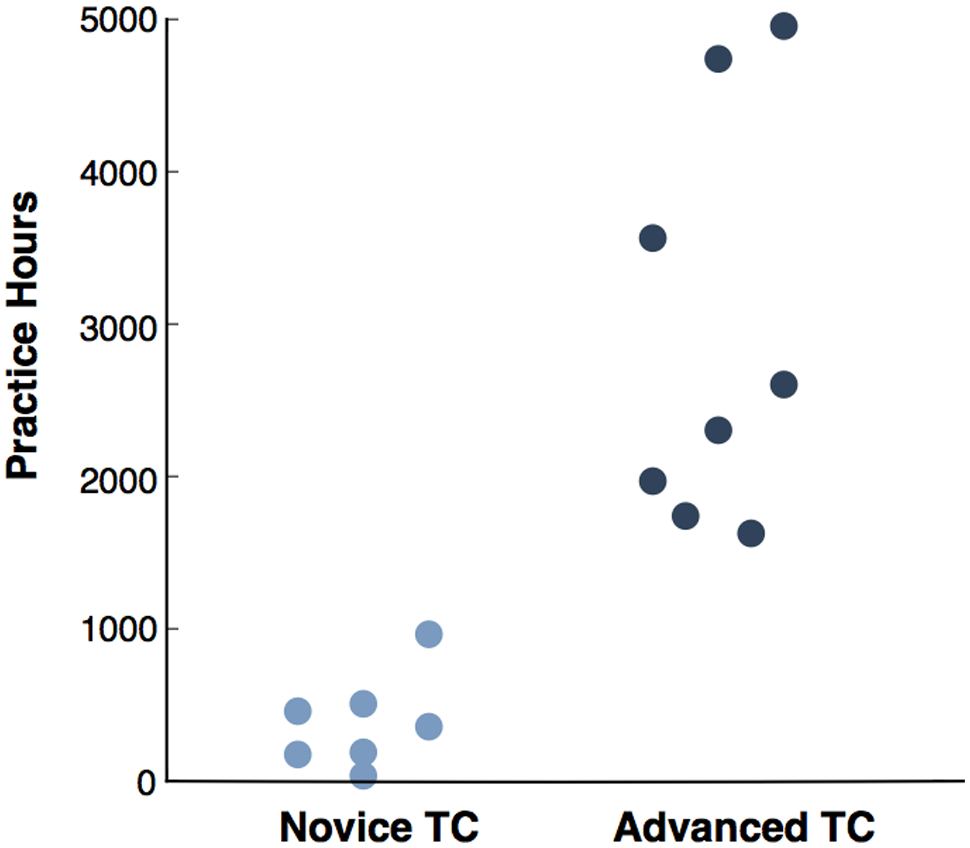
**The distribution of TC subjects’ cumulative hours of practice experience: the seven subjects whose cumulative hours fall below 1000 h (a measure which multiple studies of contemplative practice (Taylor et al., 2011; van Vugt, 2015; Zeidan, 2015) have used to divide novice from advanced practitioners) were considered novice TC, and the remaining eight practitioners were considered advanced TC**.

An important prospective design consideration for both experiments was to consider the effect of cumulative practice experience (measured as self-reported hours of practice). Previous studies have shown cumulative practice can have significant effects on both neural (Brefczynski-Lewis et al., 2007) and behavioral sensorimotor parameters (Fox et al., 2012), especially in a group such as the present sample in which there exists considerable variability in practice experience (**Figure 1**). Therefore, for study 1, we considered novice and advanced practitioners as separate groups in order to hypothesize that we would see a monotonic decrease in beta IMC with increasing experience: controls > novice > advanced (see methods for our description of criteria used to divide the TC group into novice vs. advanced TC).

For study 2, hypotheses were constructed in light of a recent study showing no effect of an embodied contemplative practice (Yoga) on the RHI (David et al., 2014). Importantly, this null finding did not consider the effect that wide variance in practice experience among the Yoga practitioners (1.5–12 years) might have had on statistical tests of between group differences. In the current investigation, rather than evaluating the difference between a control group and the TC practitioner group, the study tested correlation between cumulative TC practice hours and subjective and objective responses to the RHI. Moreover, rather than evaluating the effects of practice on the overall subjective response to the RHI, the study focused more narrowly on specifically hypothesized sub-components of the RHI response, looking specifically at the tactile and proprioceptive questionnaire items (e.g., **Table 1**, items #1 and #4) in addition to the questionnaire item related to subjective ownership responses to the illusion (**Table 1**, item #3). We hypothesized that cumulative TC practice would be associated with (1) reduced responsiveness to a subjective tactile RHI question (“How much does it seem that the touch you are feeling is where you see the rubber hand being stroked?”) (2) greater accuracy in proprioceptive judgment of the real arm through reduced objective measures of proprioceptive drift and reduced responsiveness to the subjective proprioception RHI question (“How much does it seem as though you are losing sense of where your own hand is?”) and (3) reduced perceived ownership of the rubber hand as determined by responsiveness to the subjective ownership RHI question (“How much does it seem as though the rubber hand is your hand?”). A positive finding would suggest that over time, TC teaches practitioners to maintain connection to tactile/proprioceptive bodily sensations in a manner that may inhibit visual body illusions (e.g., the RHI).

**Table 1:** Five-item questionnaire derived from (Longo et al., 2008a; Jenkinson et al., 2013) to probe subjective aspects of the RHI.

Rubber Hand Illusion Questionnaire
(1) How much does it seem that the touch you are feeling is where you see the rubber hand being stroked?
(2) How much does it seem as if you might have more than one left hand?
(3) How much does it seem as though the rubber hand is your hand?
(4) How much does it seem as though you are losing the sense of where your own hand is?
(5) How much does it seem as though you are losing the sense of owning your hand?

*Subjects respond with a number from 0 to 6 in response to the question, with lower numbers indicating negative responses and greater numbers indicating positive responses. Study hypotheses focused on TC practitioner responses to the tactile (Q1), ownership (Q3), and proprioceptive (Q4) questionnaire items.*

## Materials and Methods

### Study 1: Force Variability and Beta IMC

#### Subjects

Fifteen TC practitioners (64.3 ± 4.7 years, 9 female) from the Brookline Tai Chi studio [Brookline, MA, see (Frantzis, 2006) for details] and 16 control subjects (61.1 ± 6.0 years, 8 female) from the greater Boston, MA and Providence, RI communities were recruited to take part in this study (see **Figure 1**). Subjects were matched for age (*p* > 0.05) and education (*p* > 0.05) and were all right handed. Exclusion criteria for all subjects included the following: chronic arm, wrist, or hand pain in the last 2 years, any other movement or rheumatoid disorder, or recent muscle sprain to the hand, wrist, arm, or shoulder. TC practitioners must have maintained practice for at least three times per week for 12 months. This study was carried out in accordance with the recommendations of the Institutional Review Board at Brown University with written informed consent from all subjects. All subjects gave written informed consent in accordance with the Declaration of Helsinki.

#### Paradigm

Subjects were seated and asked to rest their right hand on a padded surface. Using their right thumb and index finger, subjects were instructed to apply a pinch grip onto a pair of compliant levers (compliance of 0.0167 N/mm) from a custom-built spring loaded lever grip device that included a load cell (A201 series FlexiForce Sensors, Tekscan, Inc. Boston, MA, USA).

**FIGURE 2:**
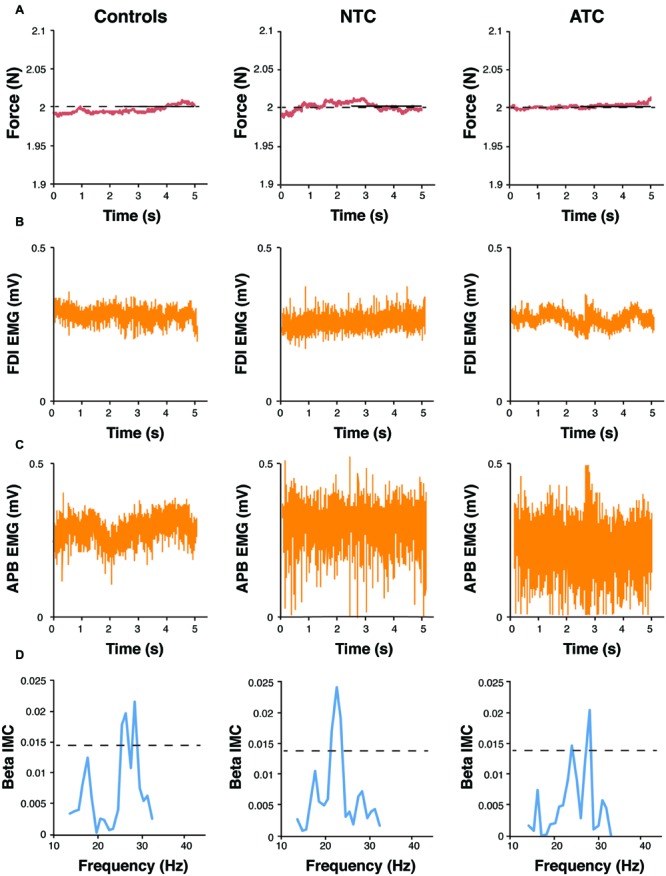
**(A)** Trace of task-related force variability in a 2-Newton force, 40 s static hold task, is displayed in a sample control, novice TC, and advanced TC subject. Surface electromyography (EMG) recorded from the **(B)** *first dorsal interosseous* (FDI) and the **(C)** *abductor pollicis brevis* (AbPB) muscles of the right hand in a sample control, novice TC, and advanced TC subject. **(D)** Examples of peak beta (15–30 Hz) intermuscular coherences (IMC) in a sample control, novice TC, and advanced TC subject.

In the task, subjects were directed to maintain a low force precision grip of 2 ± 0.03 N to the best of their ability for six separate 40-s trials while being provided with real time visual feedback of their applied force on a computer monitor. In three of the six trials, subjects were asked to attend to the sensory experience of their thumb and index finger, and in the remaining trials no explicit instructions were given (Note: one serious limitation of this approach was that there was no measurement of the efficacy of the subject’s attentional shift). Time was kept by an experimentalist, and all trials were monitored for performance. Trials with significant fluctuations in force were excluded in the analysis. Subjects were also initially granted one practice trial to become familiar with the task. An example of the force trace can be seen in **Figure 2**.

#### Recordings

Surface electromyography (EMG) was recorded from the first dorsal interosseous (FDI) and the abductor pollicis brevis (AbPB) muscles of the right hand. The electrodes were placed on the belly of each muscle and placement accuracy was assessed using visual inspection of the raw output. EMG signals were amplified and band-pass filtered at 1–200 Hz. Both the EMG and the force readings were digitized with an analog-to-digital converter (Labchart, AD Instruments) at 1,000 samples/s. Prior to analysis, EMG signals and force readings were notch filtered at 60 Hz. Sample EMG traces can be seen in **Figure 2**.

#### Median Split of TC Group into Novice and Advanced Practitioners

An important part of our analysis was to consider prospectively the effects of cumulative TC experience on beta IMC. The basis of this focus on experience comes from several previous studies reporting significant differences even within training groups, specifically between novice and advanced practitioners. This suggests that a simple comparison between controls and TC practitioners would obscure potential relationships. A single test comparing controls, novice practitioners, and advanced practitioners allows us to evaluate both the effects of differences between TC and controls as well as between novice practitioners and advanced practitioners, while at the same time minimizing the number of required statistical tests.

Therefore, the study used a median split to divide the TC group in half in order to test the effect of practice (see **Figure 1**). A simple division of subjects into three groups (controls, novice TC practitioners, and advanced TC practitioners) allowed us to evaluate the effects of TC practice in relation to two questions: (1) do novice TC practitioners manifest lower beta IMC than controls? and (2) do advanced TC practitioners manifest lower beta IMC than novice TC practitioners?

As there were an odd number of TC subjects (*n* = 15), we took a principled approach to setting the parameters of the median split. The distribution of subject hours showed that the seven subjects with lowest hours fell well below 1000 h, which is a cut-off that multiple studies of contemplative practice (Taylor et al., 2011; van Vugt, 2015; Zeidan, 2015) have identified as an important cut point separating two distinct, differentiable groups (i.e., novices vs. advanced). By contrast, the next highest subject (subject 8) reported 1626 practice hours. Thus, based on the principle that expertise emerges in practitioners with more than 1000 h of practice, the TC group was split into seven novice practitioners and eight advanced practitioners.

#### Analysis

To carry out the analysis, beta IMC and force variability were computed for each of the three groups: controls (*n* = 16), novice practitioners (*n* = 7), and advanced practitioners (*n* = 8). Data from the six trials of each subject were concatenated together as no significant inter-trial differences were observed between baseline and attention trials, and the magnitude-squared-coherence between the FDI and AbPB signals was calculated using the equation and statistical significance commonly described in the literature (Halliday et al., 1995; Terry and Griffin, 2008). Specifically, a 1024 sample window (frequency resolution = 0.977 Hz), Hanning taper, and 50% overlap were used on de-trended and rectified EMG signals (see **Figure 2** for EMGs and peak beta IMC for typical subjects in each group). Peak coherence values in the beta band (15–30 Hz) were obtained as arc hyperbolic tangent transformed as in (Halliday et al., 1995; Johnson and Shinohara, 2012).

Force variability was calculated as the root mean square of the absolute difference between the subject’s applied force and the constant 2 N target force as in (Witte et al., 2007). This measure provides a reliable indicator of the error between the target force and the applied force, and therefore a measure of the variability with which subjects could maintain a static hold.

**FIGURE 3:**
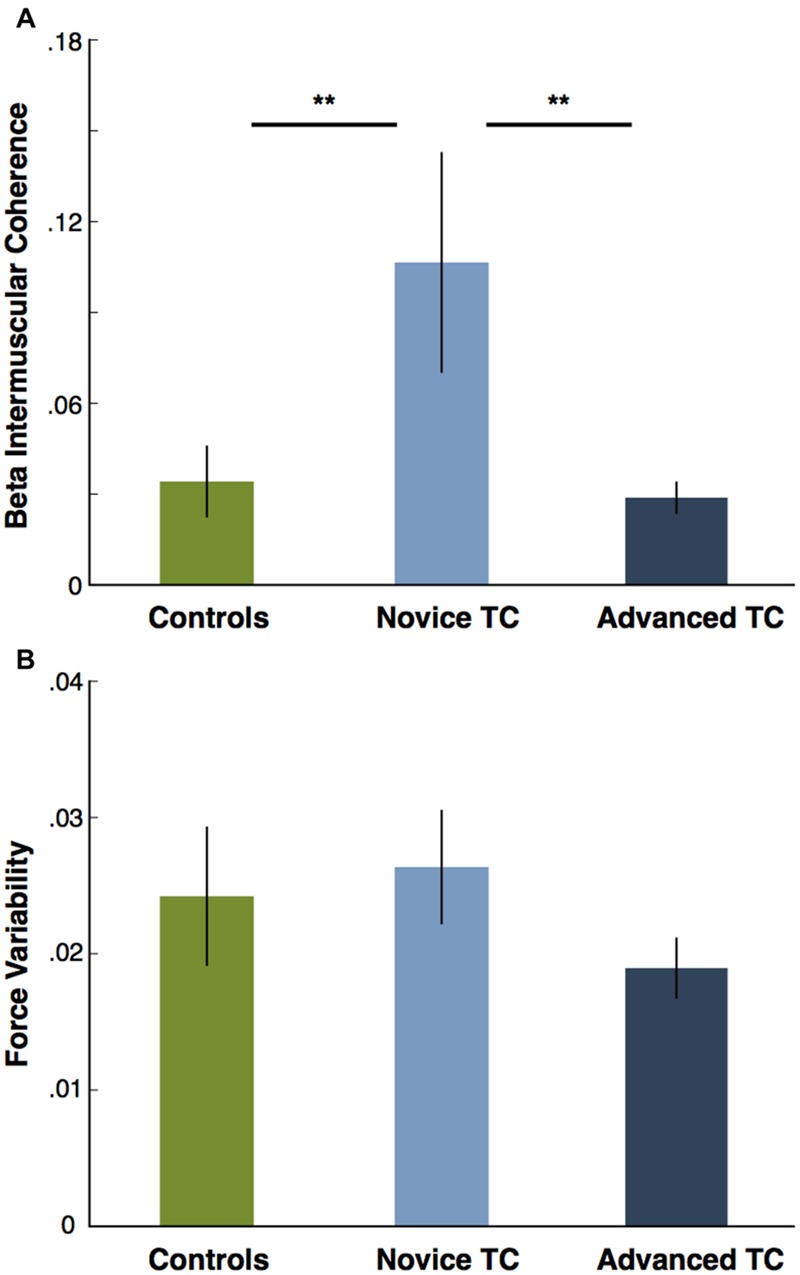
**(A)** Comparison of beta IMC across three groups (controls, novice practitioners, and advanced practitioners) shows novice TC have significantly higher beta IMC than advanced practitioners (^∗∗^*p* < 0.005) and controls (^∗∗^*p* < 0.005) while there is no difference between controls and entire TC group or between controls and advanced TC. **(B)** Comparison of force variability across three groups (controls, novice practitioners, and advanced practitioners) shows no significant differences between groups (*p* > 0.05), although advanced practitioners manifest a trend toward a significant difference as compared with novice practitioners (*p* < 0.1). In both plots, error bars display ±1 SEM.

**FIGURE 4:**
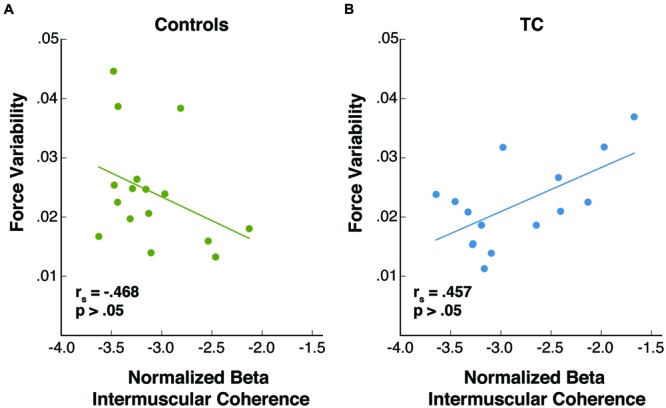
**Relationship between beta IMC and force variability in Controls and TC subjects **(A)** TC subjects showed no significant correlation between beta IMC and force variability (Spearman’s *rho* = 0.457, *p* > 0.05) although a trend was present (*p* < 0.1). (B)** Control subjects showed no significant correlation between beta IMC and force variability (Spearman’s *rho* = -0.468, *p* > 0.05) although a trend was present (*p* < 0.1). The difference between the two correlation coefficients is significant (*p* < 0.01). Note: for purposes of display, data are shown here as log-transformed, while all statistical analysis was performed on non-transformed data (since tests used were non-parametric).

Because of the study’s small sample sizes all statistical tests were performed using non-parametric statistics as suggested by Mehta and Patel (1999). A further rationale for non-parametric tests comes from the fact that beta IMC has a highly non-normal distribution (determined in our sample with the Lilliefors test), as is commonly observed in the literature (Kilner et al., 1999; Johnson and Shinohara, 2012). Thus, the non-parametric Kruskal–Wallis test was used to compare beta IMC and force variability in controls, novice practitioners, and advanced practitioners (see **Figure 3**). The Spearman correlation coefficient was used to assess the relationship between force variability and beta IMC (see **Figure 4**). Because an important element of our hypothesis concerns the difference in the relationship between beta IMC and force variability in TC versus control subjects, we also evaluated the difference between Spearman correlation coefficients between the two groups in their respective correlations of beta IMC and force variability using procedures derived from (Raghunathan et al., 1996; see **Figure 4**). To summarize the statistical plan for study 1, we first carried out a 1-way test of significance for the three-group comparison using a non-parametric Kruskal–Wallis with the plan, in the case of significance, of following up with paired comparisons, using the non-parametric Mann–Whitney test to conduct the following tests: Control vs. Novice TC, Control vs. Advanced TC, and Novice TC vs. Advanced TC for a total of three planned comparisons. To account for the effect of multiple tests, significance in both the beta IMC and force variability group analyses were Bonferroni corrected with significance specified as *p* = 0.0167 (e.g., 0.05/3).

### Study 2: Tactile and Proprioceptive Components of the RHI

#### Subjects

Fifteen TC practitioners (51.5 ± 14.1 years, 6 female) were recruited from the Brookline Tai Chi School in order to conduct the RHI task (Note: seven subjects in study 2 also participated in study 1) As in the beta IMC study, exclusion criteria for all subjects included the following: chronic arm, wrist, or hand pain in the last 2 years; any other movement or rheumatoid disorder; or recent muscle sprain to the hand, wrist, arm, or shoulder. TC practitioners must have maintained practice for at least three times per week for 12 months. All subjects gave informed consent to participate in this study in accordance with the Institutional Review Board of Brown University.

#### Paradigm

Subjects were seated in front of a two-compartment box that was open on the front and back such that they could place their hand inside the box. One compartment was covered by opaque black Plexiglas, and subjects were instructed to place their right hand inside this compartment such that it was hidden from view. The other compartment was transparent and positioned directly in front of the subject’s chest, and a realistic looking rubber arm was placed inside of it facing the same direction as the subject’s real, hidden arm, such that the rubber hand was placed eight inches laterally from the subject’s midline.

During the task, subjects were told to maintain their gaze fixed on the rubber arm. The experimenter sat across from the subject and, using both index fingers, stroked digits two to four of both the subject and the rubber arm either synchronously or asynchronously at 0.5–1 Hz. This tactile stimulation lasted 2 min, and the order of synchronous and asynchronous trials was randomized. In asynchronous trials, strokes on the real arm occurred with a 180-degree offset to strokes of the rubber arm.

Following stimulation, subjects were administered a five item questionnaire derived from (Longo et al., 2008a; Jenkinson et al., 2013), which probed subjective aspects of the illusion (see **Table 1**). The experimenter read the questions aloud and prompted subjects to respond with a number from zero to six indicating their response to the question, with lower numbers indicating negative responses and greater numbers indicating positive responses. Additionally, an objective measure of susceptibility to the illusion, proprioceptive drift, was quantified by placing a ruler extending across the two-compartment box and asking participants under which number they felt their index finger rested. In order to blind the subject to any bias about his/her sense of where the hand ought to be located, the ruler was then shifted slightly such that the numbers seen by the participant were also shifted, and the subject was again asked to judge where their index finger rested beneath the ruler. This shifting and measurement was repeated one additional time, and proprioceptive drift was calculated as the mean difference between the actual location of the participant’s index finger and the participant’s three reported values.

#### Analysis

To answer the question of whether TC cumulative practice was inversely associated with responses to somatosensory components of the RHI (e.g., tactile and proprioceptive responses) in addition to perceived illusory body ownership, we utilized a brief questionnaire assembled by Jenkinson et al. (2013) in an earlier study, focusing especially on responses to item #1 (“How much does it seem that the touch you are feeling is where you see the rubber hand being stroked?”), item #3 (“How much does it seem as the rubber hand is your hand?”), and item #4 (“How much does it seem as though you are losing sense of where your hand is?”).

**FIGURE 5:**
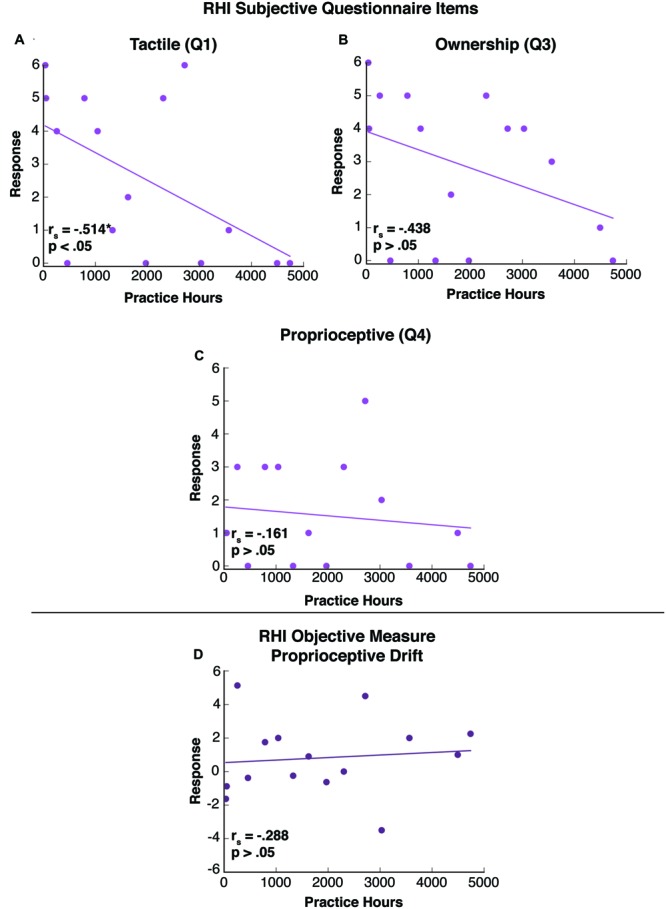
**(A)** Cumulative TC practice hours are inversely correlated with subjective – tactile response to the RHI (Response to question #1: “How much does it seem that the touch you are feeling is where you see the rubber hand being stroked?” Spearman’s *rho* = -0.514, ^∗^*p* < 0.05). **(B)** Cumulative TC practice hours are not correlated with subjective – ownership responses to the RHI (Response to question #3: “How much does it seem as the rubber hand is your hand?” Spearman’s *rho* = -0.438, *p* > 0.05). **(C)** Cumulative TC practice hours are not correlated with subjective – proprioceptive responses to the RHI (Response to question #4: “How much does it seem as though you are losing sense of where your own hand is?” Spearman’s *rho* = -0.161, *p* > 0.05). **(D)** Relationship between cumulative TC practice hours and objective measure of proprioceptive drift in response to RHI (Spearman’s *rho* = 0.288, *p* > 0.05) is non-significant.

We carried out non-parametric Spearman correlations between cumulative TC practice hours and the these three questions in the questionnaire (see **Figures 5A–C**). We also carried out a non-parametric Spearman correlation between TC practice hours and an objective measure of proprioception in the RHI, proprioceptive drift (see **Figure 5D**). In study 2, since each of the two tests reflected a separate *a priori* hypothesis that focused on a separate statistical parameter, no correction for multiple comparisons was required. Therefore statistical significance was taken to be *p* = 0.05.

## Results

### Study 1: Beta IMC

To test the hypothesis that across the three groups, increasing levels of TC experience would be associated with decreasing levels of beta IMC, we compared beta IMC in controls, novice TC, and advanced TC using the Kruskal–Wallis non-parametric one-way ANOVA, and found a significant main effect of group (*p* < 0.05; **Figure 3A**). As a follow-up, four non-parametric Mann–Whitney tests were conducted and revealed a significant difference (Bonferroni corrected to *p* = 0.0167) between novice and advanced TC (*p* < 0.005). Novice TC also showed significantly greater beta IMC than controls (*p* < 0.005), resulting in an inverted U-shaped effect of TC experience on beta IMC. As an additional comparison, using the Mann–Whitney, we also determined that there was no difference between controls and the overall TC group or between controls and advanced TC (*p* > 0.05).

To test the hypothesis that across the three groups, increasing levels of TC experience would be associated with decreasing levels of force variability, we compared force variability in controls, novice TC, and advanced TC using the Kruskal–Wallis non-parametric one-way ANOVA, and found no significant difference between groups (*p* > 0.05; **Figure 3B**). However, it is worth noting that advanced TC practitioners manifested a trend toward significantly reduced force variability when compared to novices (*p* < 0.1), but not controls (*p* > 0.1).

To characterize the relationship between beta IMC and force variability in controls vs. TC practitioners, we calculated the non-parametric Spearman correlation coefficient (**Figure 4**). No significant correlation was observed between beta IMC and force variability in TC subjects (Spearman’s *rho* = 0.457, *p* > 0.05) or between beta IMC and force variability in controls (Spearman’s *rho* = -0.468, *p* > 0.05). However, using an approach described in Raghunathan et al. (1996), we evaluated the statistical significance of the difference between the two correlation coefficients and found there was a significant difference between the positive correlation in TC and the negative correlation found in Controls (*p* < 0.01).

### Study 2: Tactile and Proprioceptive Components of the RHI

We examined the effects of TC practice on tactile and proprioceptive elements of the RHI. In assessing the tactile components of the illusion using a non-parametric Spearman correlation, we found an inverse relationship between cumulative TC practice hours and responses to the tactile question on our administered questionnaire (“How much does it seem that the touch you are feeling is where you see the rubber hand being stroked?”; Spearman’s *rho* = -0.514, *p* < 0.05; **Figure 5A**) with a median response of 2 and an interquartile range (IQR) from 0 to 5. We found no relationship between cumulative TC practice hours and responses to the question of body ownership (“How much does it seem as the rubber hand is your hand?”; Spearman’s *rho* = -0.438, *p* > 0.05) (**Figure 5B**) with a median response of 4 and an IQR from 0.25 to 4.75, or to the question of proprioception (“How much does it seem as though you are losing sense of where your hand is?”; Spearman’s *rho* = -0.161, *p* > 0.05; **Figure 5C**) with a median response of 1 and an IQR from 0 to 3. We also tested this correlation in an objective measure associated with proprioception, proprioceptive drift, and found there was no relationship between this metric and cumulative practice hours (Spearman’s *rho* = 0.288, *p* > 0.05; **Figure 5D**).

## Discussion

Contrary to our initial hypothesis of a monotonic decrease in beta IMC with increasing TC experience, we found an inverted U-shaped effect of TC practice on beta IMC, in which novice practitioners presented with greater beta IMC than both controls and advanced practitioners. Furthermore, we found that TC significantly altered the relationship between beta IMC and force variability in our elderly sample, although not as expected based on studies in younger, healthy subjects (where higher beta is associated with better task performance; Witte et al., 2007; Johnson and Shinohara, 2012). In addition, TC practice elicited enhanced subjective maintenance of ongoing tactile awareness during the RHI, although we did not find a direct modulation of the subjective experience of proprioception or body ownership or an objective measure of proprioceptive drift.

### The Effect of TC on Beta IMC

Our data suggest that TC affects beta IMC in a more complex manner than initially predicted. Rather than a linear decrease in beta IMC with increasing TC experience, there is an initial surge of beta IMC in novice practitioners that returns to levels equivalent to those seen in elderly controls with additional practice hours (see **Figure 3**). In this study we found that even the most inexperienced practitioners with only dozens of hours of practice showed significantly elevated beta IMC, possibly indicating that TC training elicits changes in neurophysiological function from very early in the practice. This finding suggests that neural pathways accessed by TC training may be utilized differently as practitioners’ levels of expertise increase. The timeframe of the initial increase in beta IMC seen in novices is consistent with motor benefits of TC reported in 12 weeks randomized controlled trial interventions [see, for example, (Chen et al., 2012) and these benefits have been found to be maintained in studies of long term practitioners (Tsang and Hui-Chan, 2003)]. The inverted U-shaped curve has also been observed in previous studies of contemplative practice (Brefczynski-Lewis et al., 2007) and other forms of language and motor learning (Rakisona and Yermolayevaa, 2011). However, it is still unclear why these pathways, specifically beta IMC, are modulated in this way.

One possible explanation for this observed trajectory of an initial surge followed by a decrease to normal levels in beta IMC in TC practitioners, is the increasingly replicated finding that beta oscillations and beta coherence are associated with not only motor but also sensory function. Coherent activity in the beta band has been observed between muscle spindles and cortex (Baker et al., 2006) and granger causality analyses (Brovelli et al., 2004) have revealed ascending directionality in the beta band from muscles to cortex in addition to the descending cortical to muscular control. Beta power in somatosensory cortex (Jones et al., 2010) as well as in the muscles (van Ede and Maris, 2013) has also been found to predict tactile detection. These findings support the theory that beta oscillations integrate both efferent motor and afferent sensory flows of information.

Given this emerging literature, our prior hypothesis of a monotonic decrease in beta IMC may not have accounted for beta’s sensory role. Taking this sensory role into account, the emphasis on somatic attention during movement in TC may shift the balance between efferent and afferent flows of sensorimotor information in beginners, while in advanced practitioners it may alter the functional significance of beta IMC to expand its ability to play a role in more efficiently filtering not only motor but also sensory information. Perhaps the initial rise in beta IMC in novice TC practitioners represents an effortful transformation in attending to afferent sensory experience, as opposed to motor experience as in controls, and with more practice this unique somatic attention becomes ingrained and more subtly and flexibly engages peripheral beta feedback loops. In other words, sensorimotor attention in TC may train efferent-afferent feedback loops and enhance the ability of beta oscillations to filter sensory and motor information, similar to the way mindfulness enhances the ability of alpha oscillations to filter somatosensory information in cortex (Kerr et al., 2013).

Partial support for this theory lies in the opposing correlations observed between beta IMC and force variability in our TC practitioners and controls. Although non-significant, in TC practitioners there was a trend toward lower force variability (i.e., better performance) being associated with *lower beta IMC*, while in controls the opposite relationship was observed, with a trend toward better task performance and lower force variability being associated with *higher beta IMC* (see **Figure 4**). One possible explanation for this contrast could be due to the specific manner in which TC utilizes beta IMC. In this context of TC practice, perhaps lower levels of beta IMC permit more reliable transmission of tactile and proprioceptive information, allowing advanced TC practitioners to engage with their sensory experience with heightened awareness of somatic sensation.

More rigorous studies utilizing electroencephalography (EEG), granger causality, and integrated sensory and motor tasks must be conducted to fully examine these hypotheses, but nonetheless the data presented here provide preliminary evidence for a complex effect of TC practice on neurophysiological indices underlying sensorimotor function, such as beta IMC. Additionally, the data suggests that future studies should investigate the clinical role of TC training in Parkinson’s patients, a motor disorder characterized by maladaptive beta oscillations (Jenkinson and Brown, 2011).

### The Effect of TC on the RHI

As hypothesized, we found that with greater experience, TC practitioners were less likely to misattribute the location of the tactile stimulation on their real, hidden hand to the visible, rubber hand (**Figure 5A**). This result suggests that TC practice is associated with subjective reports of enhanced ongoing awareness of one’s own tactile afferent processes, and is also consistent with previous findings of enhanced tactile acuity in TC (Kerr et al., 2008). However, we did not find a similar effect in an objective measure of proprioception, proprioceptive drift (**Figure 5D**). We also observed null findings in additional subjective measures, e.g., body ownership (**Figure 5B**) and proprioception (**Figure 5C**).

Taken together, these findings suggest that certain practices, such as TC observed here, may train distinct components of the RHI. Previous findings have already demonstrated that these components that are often considered together actually make up distinct responses that are not always directly correlated (e.g., proprioceptive drift and sense of ownership experience (Rohde et al., 2011; Abdulkarim and Ehrsson, 2015). In this study we found that TC practice exerted specific effects on tactile and not proprioceptive subjective experience in the RHI. One possible interpretation of these findings is that there may exist a tradeoff between an improved sense of proprioception and enhanced plasticity, and given prior evidence that TC practice induced plastic changes in specifically tactile acuity (Kerr et al., 2008), our sample of TC practitioners may lack proprioceptive benefits. Additionally, as predicted by Bayesian causal inference models (Kilteni et al., 2015; Samad et al., 2015) this increased tactile awareness (or reduced perceptual error) in our sample of TC practitioners may affect the overall sense of illusory body ownership. However, more rigorous examination of the individual factors thought to underlie the RHI must be conducted in TC practitioners in order to support these claims.

The present study’s results suggest TC may alter the RHI in highly specific and subtle ways that an undifferentiated approach to the RHI may miss. The specificity of this data may also explain the negative finding seen in a prior study of RHI and yoga (David et al., 2014), which did not consider the different components of the illusion separately but instead evaluated yoga’s effect on a compounded, scored measure. Additional future directions should include studies correlating basic sensorimotor beta rhythm network coherence and higher-order RHI in order to more fully understand the possible relation between these two variables. Early reports suggest beta rhythm transmission from motor areas may enable the multisensory perceptual processes that underlie the RHI [see for example (Mancini et al., 2013)].

Despite their exploratory nature, these data may provide some clues about how to model sensorimotor mechanisms underlying TC’s efficacy in enhancing sensorimotor function, especially in the elderly. Specifically, these data suggest that, given the strong difference we found between novices and advanced TC beta IMC, there may be developmental phases over the course of TC practice such that novices may be using very different sensorimotor neural processes than those who have undertaken more than 1000 h of practice. More data collected on the behavioral changes and experiential self-reports of practitioners across the developmental span of expertise would be very helpful. Moreover, what the RHI data suggest is that there may be definitive shifts across this developmental span in which practitioners undergo changes in “embodiment” e.g., enhanced sensory and motor processing elicited by increased somatic attention during practice. In other words, practitioners’ experiences of somatic attention and sensory and motor performances may shift over time; the current study’s focus on the significant effects of practice over time may shed light on TC as a modality for retraining basic sensorimotor neural substrates (e.g., beta rhythm network coherence) and higher-order multisensory body representation (e.g., specific aspects of the RHI).

Considered from a more theoretical perspective, the correlation of TC practice with the self-reported maintenance of tactile awareness during the RHI provides partial support for an intriguing hypothesis derived from some sources in the TC instructional literature (Wayne and Fuerst, 2013). The hypothesis states that over time, cumulative TC practice may mechanistically move practitioners toward a more unified, less fragmented and more “spacious” sense of their own internal bodily experience, which is sometimes described by practitioners’ use of highly qualitative words such as “presence” or a sense of energy in the body, sometimes referred to by the Chinese term “qi” (Yang, 2005). The broader implications of these ideas are beyond the scope of this exploratory study. However, further investigation of the experience of TC practitioners, utilizing qualitative tools recently used in studies of the phenomenology of the RHI (Valenzuela Moguillansky et al., 2013) could be a productive and important avenue for future research.

### Limitations

This study has several important limitations, some of which are quite prominent.

First, in study 1, the data are cross-sectional so self-selection confounds and spurious causative agents cannot be ruled out and although there is an age-matched control group, the study does not use the highest type of control condition which would also control for practice-related activity (e.g., would compare TC to some other type of activity-focused group such as “tango dancers” in order to rule out the effects of group-related physical and mental practice activity). In addition, it is important to note that the samples sizes are also too small in our study of beta IMC and force variability (*n* = 31) and the RHI (*n* = 15) to draw definitive conclusions and should be replicated in larger samples.

Second, an important limitation in our study of beta IMC is that beta IMC is not identical to beta CMC, although the two measures are highly related (Kilner et al., 1999; van Ede and Maris, 2013). In order to adequately investigate the involvement of cortex in the hypothesized body awareness mechanism a more rigorous methodology using EEG-EMG and directed coherence analyses between primary somatosensory cortex, primary motor cortex, and the muscles must be conducted.

Third, a key limitation in our investigation of the RHI is that we did not compare TC with a control group so we cannot say whether TC practice is associated with decreased subjective tactile responses to the RHI when comparing TC practitioners to normal controls.

## Conclusion

We found that TC practice modulates beta IMC in an inverted U – shaped trajectory, where novice TC practitioners manifest a sharp increase in beta IMC as compared with controls that with increasing practice again returns to levels equivalent to controls as practitioners become more advanced. This finding suggests that TC practice elicits complex changes in sensory and motor processes over the developmental lifespan of TC training. Additionally, the inverse association between beta IMC and force variability typically observed in healthy, younger populations actually showed a trend in the opposite direction in our TC population, such that higher levels of beta IMC actually indicated higher force variability (i.e., worse task performance) in this group. Finally, we found that with increasing experience, TC practitioners were less likely to misattribute a touch on their hand during the RHI to the fake rubber hand. At the same time, however, we found no significant relationship between cumulative TC experience and proprioceptive drift or sense of body ownership.

While the results of this investigation must be approached with caution, given that the two experiments used cross-sectional design, small sample sizes, and based all analysis on EMG measures, this study provides preliminary validation for a theoretical model in which TC practice enhances filtering of sensorimotor information through peripheral feedback beta rhythm efferent-afferent loops and alters awareness of tactile sensations during the RHI. This work is the first to examine TC practice at not only the level of sensorimotor information processing but also the level of integrated body awareness. The findings presented here may help to shed light on the mechanisms underlying the widespread benefits observed with TC in symptoms associated with aging and difficult illnesses such as Parkinson’s disease.

## Author Contributions

All authors listed designed and performed research, and CK and UA analyzed data and wrote the paper.

## Conflict of Interest Statement

The authors declare that the research was conducted in the absence of any commercial or financial relationships that could be construed as a potential conflict of interest.

## References

[B1] Abdulkarim Z. Ehrsson H. H. (2015). No causal link between changes in hand position sense and feeling of limb ownership in the rubber hand illusion. *Atten. Percept. Psychophys.* 10.3758/s13414-015-1016-0 [Epub ahead of print]. PMC4744264 26555651

[B2] Baker S. N. (2007). Oscillatory interactions between sensorimotor cortex and the periphery. *Curr. Opin. Neurobiol.* 17 649–655. 10.1016/j.conb.2008.01.007 18339546 PMC2428102

[B3] Baker S. N. Chiu M. Fetz E. E. (2006). Afferent encoding of central oscillations in the monkey arm. *J. Neurophysiol.* 95 3904–3910. 10.1152/jn.01106.2005 16709725

[B4] Botvinick M. Cohen J. (1998). Rubber hands ‘feel’ touch that eyes see. *Nature* 391 756 10.1038/35784 9486643

[B5] Brefczynski-Lewis J. A. Lutz A. Schaefer H. S. Levinson D. B. Davidson R. J. (2007). Neural correlates of attentional expertise in long-term meditation practitioners. *Proc. Natl. Acad. Sci. U.S.A.* 104 11483–11488. 10.1073/pnas.0606552104 17596341 PMC1903340

[B6] Brovelli A. Ding M. Ledberg A. Chen Y. Nakamura R. Bressler S. L. (2004). Beta oscillations in a large-scale sensorimotor cortical network: directional influences revealed by Granger causality. *Proc. Natl. Acad. Sci. U.S.A.* 101 9849–9854. 10.1073/pnas.0308538101 15210971 PMC470781

[B7] Chen Y. S. Crowley Z. Zhou S. Cartwright C. (2012). Effects of 12-week Tai Chi training on soleus H-reflex and muscle strength in older adults: a pilot study. *Eur. J. Appl. Physiol.* 112 2363–2368. 10.1007/s00421-011-2182-y 21947456

[B8] Christou E. A. Yang Y. Rosengren K. S. (2003). Taiji training improves knee extensor strength and force control in older adults. *J. Gerontol. A Biol. Sci. Med. Sci.* 58 763–766. 10.1093/gerona/58.8.M763 12902537

[B9] David N. Fiori F. Aglioti S. M. (2014). Susceptibility to the rubber hand illusion does not tell the whole body-awareness story. *Cogn. Affect. Behav. Neurosci.* 14 297–306. 10.3758/s13415-013-0190-6 23888383

[B10] Engel A. K. Fries P. (2010). Beta-band oscillations–signalling the status quo? *Curr. Opin. Neurobiol.* 20 156–165. 10.1016/j.conb.2010.02.015 20359884

[B11] Eshkevari E. Rieger E. Longo M. R. Haggard P. Treasure J. (2012). Increased plasticity of the bodily self in eating disorders. *Psychol. Med.* 42 819–828. 10.1017/S0033291711002091 22017964

[B12] Fox K. C. Zakarauskas P. Dixon M. Ellamil M. Thompson E. Christoff K. (2012). Meditation experience predicts introspective accuracy. *PLoS ONE* 7:e45370 10.1371/journal.pone.0045370 PMC3458044 23049790

[B13] Frantzis B. (2006). *Tai Chi: Health for Life*. Fairfax, CA: Energy Arts.

[B14] Halliday D. M. Rosenberg J. R. Amjad A. M. Breeze P. Conway B. A. Farmer S. F. (1995). A framework for the analysis of mixed time series/point process data–theory and application to the study of physiological tremor, single motor unit discharges and electromyograms. *Prog. Biophys. Mol. Biol.* 64 237–278. 10.1016/S0079-6107(96)00009-0 8987386

[B15] Jenkinson N. Brown P. (2011). New insights into the relationship between dopamine, beta oscillations and motor function. *Trends Neurosci.* 34 611–618. 10.1016/j.tins.2011.09.003 22018805

[B16] Jenkinson P. M. Haggard P. Ferreira N. C. Fotopoulou A. (2013). Body ownership and attention in the mirror: insights from somatoparaphrenia and the rubber hand illusion. *Neuropsychologia* 51 1453–1462. 10.1016/j.neuropsychologia.2013.03.029 23603022

[B17] Jha A. P. Krompinger J. Baime M. J. (2007). Mindfulness training modifies subsystems of attention. *Cogn. Affect. Behav. Neurosci.* 7 109–119. 10.3758/CABN.7.2.109 17672382

[B18] Jha A. P. Stanley E. A. Kiyonaga A. Wong L. Gelfand L. (2010). Examining the protective effects of mindfulness training on working memory capacity and affective experience. *Emotion* 10 54–64. 10.1037/a0018438 20141302

[B19] Johnson A. N. Shinohara M. (2012). Corticomuscular coherence with and without additional task in the elderly. *J. Appl. Physiol. (1985)* 112 970–981. 10.1152/japplphysiol.01079.2011 22223451

[B20] Jones S. R. Kerr C. E. Wan Q. Pritchett D. L. Hamalainen M. Moore C. I. (2010). Cued spatial attention drives functionally relevant modulation of the mu rhythm in primary somatosensory cortex. *J. Neurosci.* 30 13760–13765. 10.1523/JNEUROSCI.2969-10.2010 20943916 PMC2970512

[B21] Jones S. R. Pritchett D. L. Sikora M. A. Stuﬄebeam S. M. Hämäläinen M. Moore C. I. (2009). Quantitative analysis and biophysically realistic neural modeling of the MEG mu rhythm: rhythmogenesis and modulation of sensory-evoked responses. *J. Neurophysiol.* 102 3554–3572. 10.1152/jn.00535.2009 19812290 PMC2804421

[B22] Kerr C. E. Jones S. R. Wan Q. Pritchett D. L. Wasserman R. H. Wexler A. (2011). Effects of mindfulness meditation training on anticipatory alpha modulation in primary somatosensory cortex. *Brain Res. Bull.* 85 96–103. 10.1016/j.brainresbull.2011.03.026 21501665

[B23] Kerr C. E. Sacchet M. D. Lazar S. W. Moore C. I. Jones S. R. (2013). Mindfulness starts with the body: somatosensory attention and top-down modulation of cortical alpha rhythms in mindfulness meditation. *Front. Hum. Neurosci.* 7:12 10.3389/fnhum.2013.00012 PMC3570934 23408771

[B24] Kerr C. E. Shaw J. R. Wasserman R. H. Chen V. W. Kanojia A. Bayer T. (2008). Tactile acuity in experienced Tai Chi practitioners: evidence for use dependent plasticity as an effect of sensory-attentional training. *Exp. Brain Res.* 188 317–322. 10.1007/s00221-008-1409-6 18512052 PMC2795804

[B25] Kilner J. M. Baker S. N. Salenius S. Jousmaki V. Hari R. Lemon R. N. (1999). Task-dependent modulation of 15-30 Hz coherence between rectified EMGs from human hand and forearm muscles. *J. Physiol.* 516(Pt 2) 559–570. 10.1111/j.1469-7793.1999.0559v.x 10087353 PMC2269269

[B26] Kilteni K. Maselli A. Kording K. P. Slater M. (2015). Over my fake body: body ownership illusions for studying the multisensory basis of own-body perception. *Front. Hum. Neurosci.* 9:141 10.3389/fnhum.2015.00141 PMC4371812 25852524

[B27] Li F. Harmer P. Fitzgerald K. Eckstrom E. Stock R. Galver J. (2012). Tai chi and postural stability in patients with Parkinson’s disease. *N. Engl. J. Med.* 366 511–519. 10.1056/NEJMoa1107911 22316445 PMC3285459

[B28] Li J. X. Xu D. Q. Hong Y. (2008). Effects of 16-week Tai Chi intervention on postural stability and proprioception of knee and ankle in older people. *Age Ageing* 37 575–578. 10.1093/ageing/afn109 18541612

[B29] Longo M. R. Cardozo S. Haggard P. (2008a). Visual enhancement of touch and the bodily self. *Conscious. Cogn.* 17 1181–1191. 10.1016/j.concog.2008.01.001 18294867

[B30] Longo M. R. Schuur F. Kammers M. P. Tsakiris M. Haggard P. (2008b). What is embodiment? A psychometric approach. *Cognition* 107 978–998. 10.1016/j.cognition.2007.12.004 18262508

[B31] MacLean K. A. Ferrer E. Aichele S. R. Bridwell D. A. Zanesco A. P. Jacobs T. L. (2010). Intensive meditation training improves perceptual discrimination and sustained attention. *Psychol. Sci.* 21 829–839. 10.1177/0956797610371339 20483826 PMC3132583

[B32] Mancini F. Longo M. R. Canzoneri E. Vallar G. Haggard P. (2013). Changes in cortical oscillations linked to multisensory modulation of nociception. *Eur. J. Neurosci.* 37 768–776. 10.1111/ejn.12080 23216684

[B33] Mehta C. R. Patel N. R. (1999). “Exact permutational inference for categorical and nonparametric data,” in *Statistical Strategies for Small Sample Research* ed. Hoyle R. H. (Thousand Oaks, CA: Sage) 1–29.

[B34] Mirams L. Poliakoff E. Brown R. J. Lloyd D. M. (2013). Brief body-scan meditation practice improves somatosensory perceptual decision making. *Conscious. Cogn.* 22 348–359. 10.1016/j.concog.2012.07.009 22889642

[B35] Moseley G. L. Gallace A. Spence C. (2012). Bodily illusions in health and disease: physiological and clinical perspectives and the concept of a cortical ‘body matrix.’ *Neurosci. Biobehav. Rev.* 36 34–46. 10.1016/j.neubiorev.2011.03.013 21477616

[B36] Moseley G. L. Olthof N. Venema A. Don S. Wijers M. Gallace A. (2008). Psychologically induced cooling of a specific body part caused by the illusory ownership of an artificial counterpart. *Proc. Natl. Acad. Sci. U.S.A.* 105 13169–13173. 10.1073/pnas.0803768105 18725630 PMC2529116

[B37] Mrazek M. D. Franklin M. S. Phillips D. T. Baird B. Schooler J. W. (2013). Mindfulness training improves working memory capacity and GRE performance while reducing mind wandering. *Psychol. Sci.* 24 776–781. 10.1177/0956797612459659 23538911

[B38] Raghunathan T. E. Rosenthal R. Rubin D. B. (1996). Comparing correlated but nonoverlapping correlations. *Psychol. Methods* 1 178–193. 10.1037/1082-989X.1.2.178

[B39] Rakisona D. H. Yermolayevaa Y. (2011). How to identify a domain-general learning mechanism when you see one. *J. Cogn. Dev.* 12 134–158. 10.1080/15248372.2010.535228

[B40] Richerson S. Rosendale K. (2007). Does Tai Chi improve plantar sensory ability? A pilot study. *Diabetes Technol. Ther.* 9 276–286. 10.1089/dia.2006.0033 17561798

[B41] Rohde M. Di Luca M. Ernst M. O. (2011). The Rubber Hand Illusion: feeling of ownership and proprioceptive drift do not go hand in hand. *PLoS ONE* 6:e21659 10.1371/journal.pone.0021659 PMC3125296 21738756

[B42] Samad M. Chung A. J. Shams L. (2015). Perception of body ownership is driven by Bayesian sensory inference. *PLoS ONE* 10:e0117178 10.1371/journal.pone.0117178 PMC4320053 25658822

[B43] Schleicher M. M. Wedam L. Wu G. (2012). Review of Tai Chi as an effective exercise on falls prevention in elderly. *Res. Sports Med.* 20 37–58. 10.1080/15438627.2012.634697 22242736

[B44] Taylor V. A. Grant J. Daneault V. Scavone G. Breton E. Roffe-Vidal S. (2011). Impact of mindfulness on the neural responses to emotional pictures in experienced and beginner meditators. *Neuroimage* 57 1524–1533. 10.1016/j.neuroimage.2011.06.001 21679770

[B45] Terry K. Griffin L. (2008). How computational technique and spike train properties affect coherence detection. *J. Neurosci. Methods* 168 212–223. 10.1016/j.jneumeth.2007.09.014 17976736 PMC2268650

[B46] Thakkar K. N. Nichols H. S. Mcintosh L. G. Park S. (2011). Disturbances in body ownership in schizophrenia: evidence from the rubber hand illusion and case study of a spontaneous out-of-body experience. *PLoS ONE* 6:e27089 10.1371/journal.pone.0027089 PMC3205058 22073126

[B47] Tsang W. W. Hui-Chan C. W. (2003). Effects of tai chi on joint proprioception and stability limits in elderly subjects. *Med. Sci. Sports Exerc.* 35 1962–1971. 10.1249/01.MSS.0000099110.17311.A2 14652489

[B48] Valenzuela Moguillansky C. O’regan J. K. Petitmengin C. (2013). Exploring the subjective experience of the “rubber hand” illusion. *Front. Hum. Neurosci.* 7:659 10.3389/fnhum.2013.00659 PMC3805941 24167480

[B49] van Ede F. Maris E. (2013). Somatosensory demands modulate muscular Beta oscillations, independent of motor demands. *J. Neurosci.* 33 10849–10857. 10.1523/JNEUROSCI.5629-12.2013 23804105 PMC6618494

[B50] van Vugt M. K. (2015). “Cognitive benefits of mindfulness meditation,” in *Handbook of Mindfulness: Theory, Research, and Practice* eds Brown K. W. Creswell J. D. Ryan R. M. (New York, NY: Guilford).

[B51] Wayne P. M. (2013). *The Harvard Medical School Guide to Tai Chi: 12 Weeks to a Healthy Body, Strong Heart, and Sharp Mind*. Boston, MA: Shambala.

[B52] Wayne P. M. Fuerst M. L. (2013). *The Harvard Medical School Guide to Tai Chi.* Boston, MA: Shamhala Publications.

[B53] Witte M. Patino L. Andrykiewicz A. Hepp-Reymond M. C. Kristeva R. (2007). Modulation of human corticomuscular beta-range coherence with low-level static forces. *Eur. J. Neurosci.* 26 3564–3570. 10.1111/j.1460-9568.2007.05942.x 18052988

[B54] Wolf S. L. Barnhart H. X. Ellison G. L. Coogler C. E. (1997). The effect of Tai Chi Quan and computerized balance training on postural stability in older subjects. Atlanta FICSIT Group. Frailty and Injuries: Cooperative Studies on Intervention Techniques. *Phys. Ther.* 77 371–381; discussion 382–374. 9105340 10.1093/ptj/77.4.371

[B55] Yang Y. (2005). *Taijiquan: the Art of Nurturing, the Science of Power*. Champaign, IL: Zhenwu Publications.

[B56] Zeidan F. (2015). “The neurobiology of mindfulness meditation,” in *Handbook of Mindfulness: Theory, Research, and Practice* eds Brown K. W. Creswell J. D. Ryan R. M. (New York, NY: Guilford).

[B57] Zeller D. Litvak V. Friston K. J. Classen J. (2015). Sensory processing and the rubber hand illusion–an evoked potentials study. *J. Cogn. Neurosci.* 27 573–582. 10.1162/jocn_a_00705 25170795

[B58] Zopf R. Harris J. A. Williams M. A. (2011). The influence of body-ownership cues on tactile sensitivity. *Cogn. Neurosci.* 2 147–154. 10.1080/17588928.2011.578208 24168529

